# Retinal Ganglion Cell Dendritic Atrophy in DBA/2J Glaucoma

**DOI:** 10.1371/journal.pone.0072282

**Published:** 2013-08-19

**Authors:** Pete A. Williams, Gareth R. Howell, Jessica M. Barbay, Catherine E. Braine, Gregory L. Sousa, Simon W. M. John, James E. Morgan

**Affiliations:** 1 School of Optometry and Vision Sciences, Cardiff University, Cardiff, United Kingdom; 2 The Jackson Laboratory, Bar Harbor, Maine, United States of America; 3 The Howard Hughes Medical Institute, Bar Habor, Maine, United States of America; 4 Department of Ophthalmology, Tufts University of Medicine, Boston, Massachusetts, United States of America; 5 Cardiff Eye Unit, University Hospital of Wales, Cardiff, United Kingdom; Hanson Institute, Australia

## Abstract

Glaucoma is a complex disease affecting an estimated 70 million people worldwide, characterised by the progressive degeneration of retinal ganglion cells and accompanying visual field loss. The common site of damage to retinal ganglion cells is thought to be at the optic nerve head, however evidence from other optic neuropathies and neurodegenerative disorders suggests that dendritic structures undergo a prolonged period of atrophy that may accompany or even precede soma loss and neuronal cell death. Using the DBA/2J mouse model of glaucoma this investigation aims to elucidate the impact of increasing intraocular pressure on retinal ganglion cell dendrites using DBA/2J mice that express YFP throughout the retinal ganglion cells driven by Thy1 (*DBA/2J.Thy1*(*YFP*)) and DiOlistically labelled retinal ganglion cells in DBA/2J mice. Here we show retinal ganglion cell dendritic degeneration in DiOlistically labelled DBA/2J retinal ganglion cells but not in the *DBA/2J.Thy1*(*YFP*) retinal ganglion cells suggesting that a potential downregulation of Thy1 allows only ‘healthy’ retinal ganglion cells to express YFP. These data may highlight alternative pathways to retinal ganglion cell loss in DBA/2J glaucoma.

## Introduction

Glaucoma is a complex multifactorial disease, which affects an estimated 70 million people worldwide. It is characterised by the selective and progressive loss of retinal ganglion cells (RGCs) and associated reduction in visual field [1], [2], [3], [4], [5], [6], [7]. Increasing age and elevated intraocular pressure (IOP) are the principle risk factors.

Several lines of experimental enquiry have confirmed that the optic nerve head is a site of early damage. These have been confirmed in human glaucoma, in animals with a scleral lamina (non human primates, NHP) and those with a glial lamina [8], [9] consistent with the hypothesis that axonal damage is the principle driver of retinal ganglion cell loss. The relationship of axonal damage to events at the cell soma and RGC synaptic connectivity has been a topic of recent study. Analyses of retinal ganglion cell morphology in NHP, feline and rodent glaucoma [10], [11], [12] have consistently demonstrated the presence of dendritic pruning prior to the loss of cell bodies. There has been considerable interest in the use of animal models to study RGC degenerative events following axon trauma where dendritic atrophy has been followed with *in vivo* imaging in mice expressing Thy1-YFP in RGCs [13]. In view of these findings, there is a pressing need to determine whether dendritic atrophy occurs in murine models of glaucoma. Given the flexibility of the mouse for genetic studies, murine models offer the greatest opportunity to determine the mechanisms underlying these degenerative changes and their relationship to axonal damage.

The DBA/2J strain of mice is one of the most widely used in glaucoma research [8], [14], [15], [16], [17], [18], [19], [20], [21], [22], [23]. DBA/2J mice inherit a disease with similarities to human pigmentary glaucoma and develop iris pigment dispersion at ∼6 months which subsequently impacts the drainage structure in the anterior chamber in the eye leading to high IOP [14]. High IOP is evident in a large portion of DBA/2J mice at ∼9 months and approximately 70% of DBA/2J mice develop moderate to severe axon and retinal ganglion cell loss by ∼12 months. To investigate the effects chronic elevation of IOP we crossed DBA/2J with *Thy1*(YFP) to generate mice that develop spontaneous pigmentary glaucoma a subset of RGCs that express YFP in the cell soma and dendritic tree. We also labelled RGCs DiOlistically using the carbocyanine dyes, DiI and DiO [24], to control for the possibility that down regulation of Thy1 as a result of the experimental glaucoma [18] could bias the quantification of dendritic structure in surviving cells.

## Results

### DBA/2J.Thy1(YFP) mice do not demonstrate changes in dendritic structure

**Figure 1:**
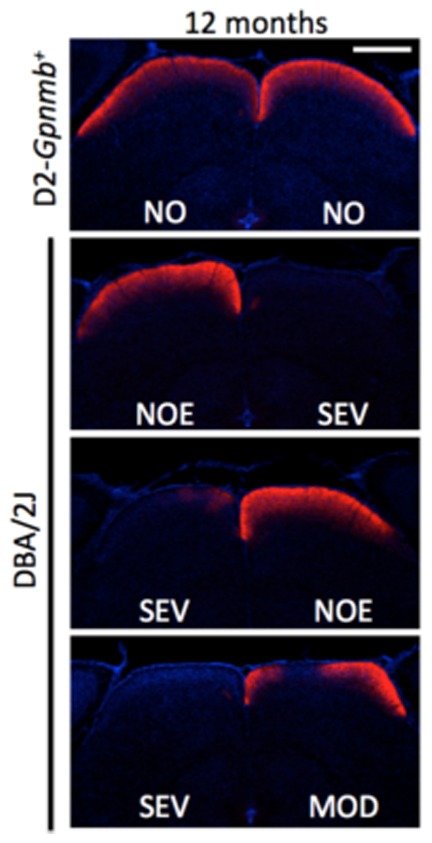
Axonal transport is normal in NOE nerves. To allow staging of dendritic changes in relation to early axon damage, axonal transport was evaluated in NOE, MOD, SEV and control eyes. There are obvious deficits in anterograde axonal transport to the SC (as assessed by fluorescent tracer) in MOD and SEV optic nerves. There was no observable difference between NOE or no glaucoma control D2-*Gpnmb* ^+^ mice at age 12 months. Scale bar  = 100 µm; NO  =  no damage.

Both eyes from DBA2J.YFP mice at 9.5–11 months were assessed by which time they would be expected to manifest retinal damage. Retinas from 1–3 month DBA/2J.*Thy1*(YFP) mice were analysed as a control group. All eyes were categorised into 3 cohorts on the basis of optic nerve damage [8]: no or early damage (NOE), moderate damage (MOD) or severe damage (SEV). Importantly, optic nerves from NOE eyes are at a very early stage of disease [18]. They are indistinguishable from optic nerves of age and sex matched control eyes and have no detectable axon loss. At this NOE stage we have not detected any evidence of dying back [8], [25], [26] and axon transport is intact ([25], (Figure 1).

Retinal ganglion cell identity was confirmed by the presence of an axon running toward the optic disc in the retinal nerve fibre layer. Typically we observed that YFP filled the terminal parts of the dendritic tree; regardless of the intensity of YFP fluorescence, we did not observe points in the dendritic tree that suggested a barrier to the distribution of YFP. In total we analysed 68 YFP positive cells from DBA/2J mice at 9.5–11 months and 35 cells from 1–3 month old mice. The mean (SD) number of cells imaged per retina was 2.59(2.33).

**Figure 2:**
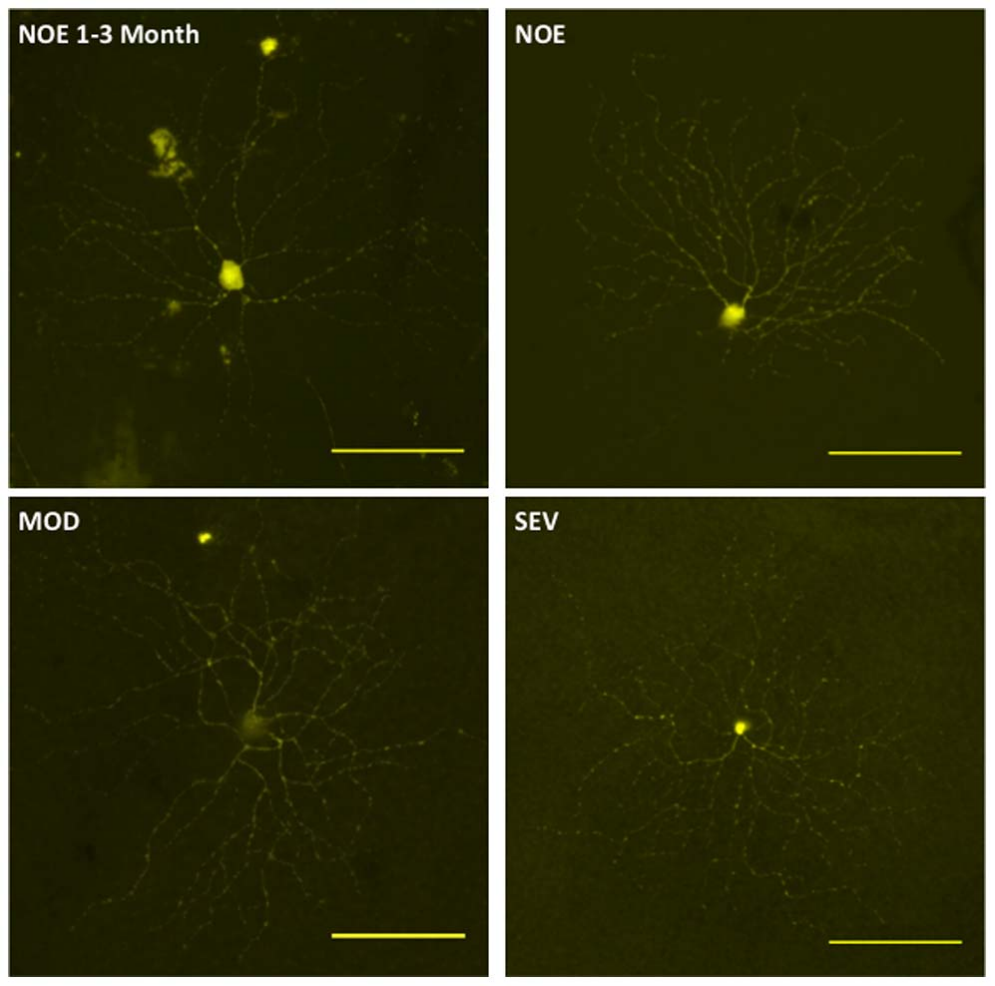
Representative retinal ganglion cells from DBA/2J.*Thy1*(YFP) mice. Panel of representative retinal ganglion cells from 1–3 month NOE (*upper left*), 9.5–11 month NOE (*upper right*), 9.5–11 month MOD (*lower left*), and 9.5–11 month SEV DBA/2J.*Thy1*(YFP) mice (*lower right*). There are no observable differences in retinal ganglion cell morphologies between groups. Scale bars  = 100 µm.

**Figure 3:**
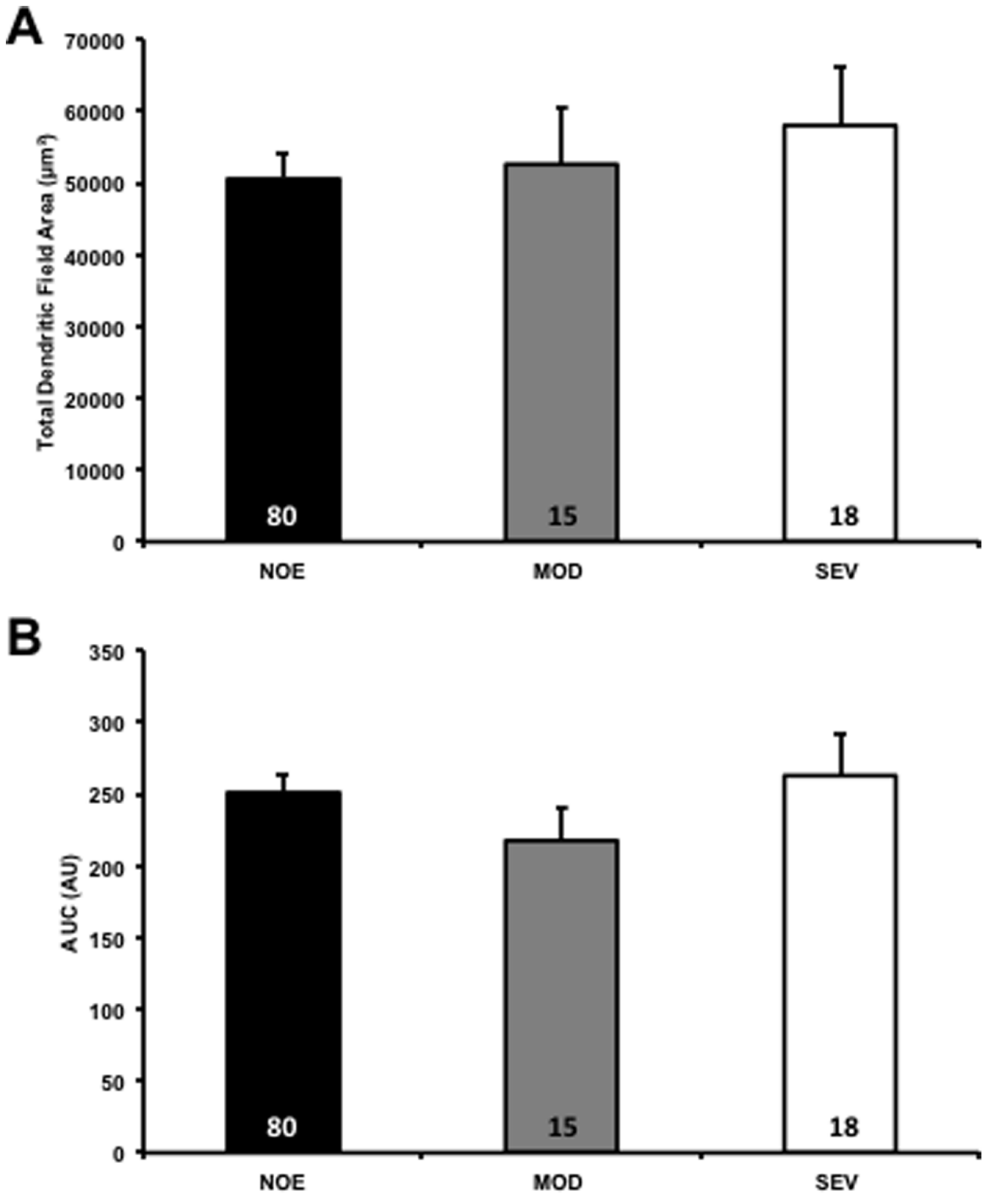
There are no significant changes to retinal ganglion cell dendritic morphology in DBA/2J.*Thy1*(YFP) mice. To explore potential changes to retinal ganglion cell dendritic morphology caused by increasing IOP and axonal damage the retinal ganglion cells of DBA/2J.*Thy1*(YFP) mice were analysed. Mice were categorised into cohorts depending on their level of optic nerve damage; no or early damage (NOE), moderate damage (MOD) or severe damage (SEV). In 9.5–11 month old DBA/2J.*Thy1*(YFP) mice there were no significant changes to retinal ganglion cells total dendritic length, total dendritic field area (A) or Sholl analysis area under the curve (AUC) (B) (*P*>0.05 across all groups). Error bars represent SEM; numbers in bars represent the *n* of cells.

**Figure 4:**
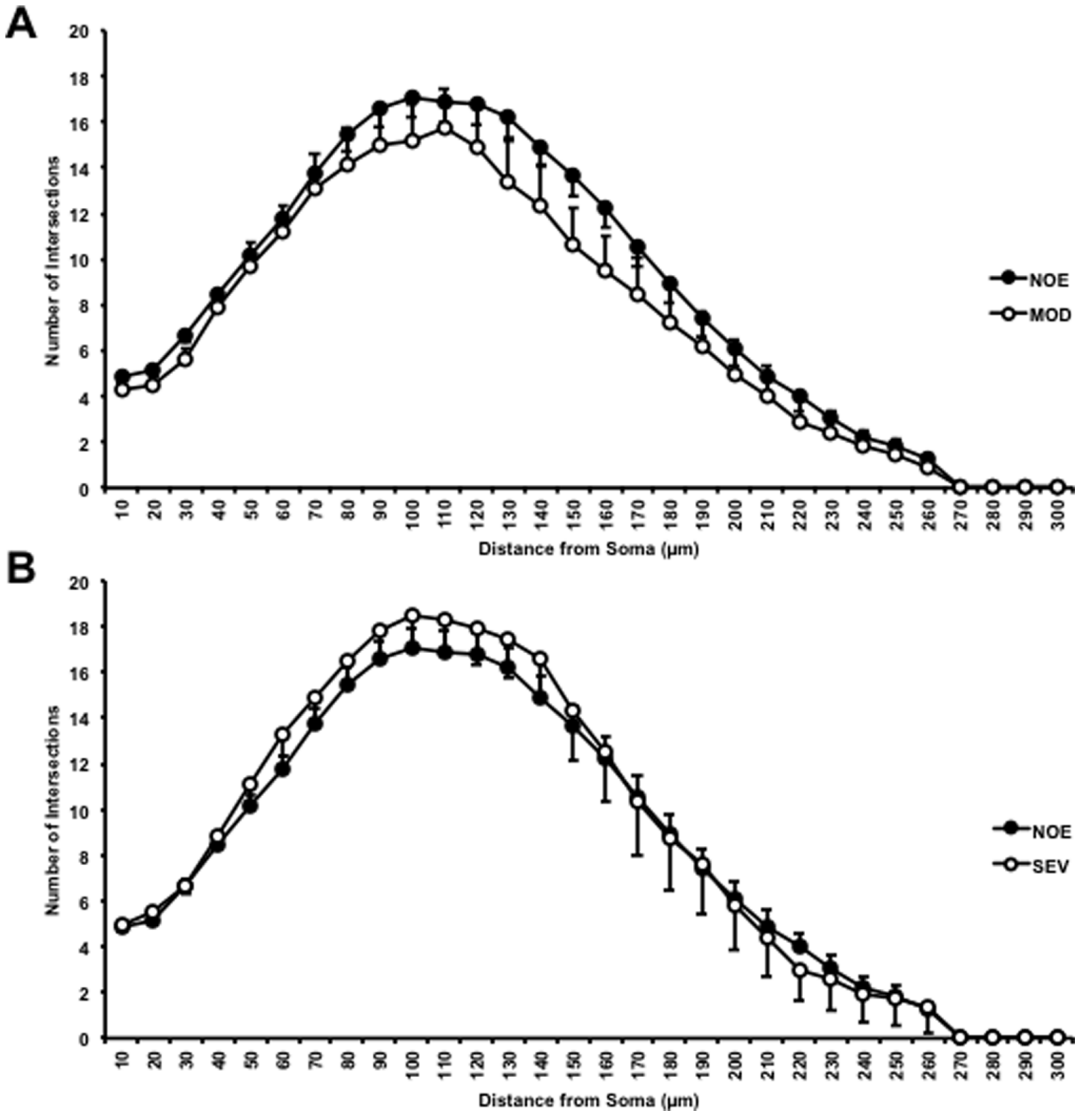
There are no significant changes to retinal ganglion cell dendritic integrity as assessed by Sholl analysis. There is no significant change in the Sholl analysis of NOE, MOD (A) or SEV (B) mouse retinal ganglion cells. Error bars represent SEM.

Representative YFP positive RGCs from glaucomatous and control eyes are shown in Figure 2. There was no significant difference in RGC dendritic morphology comparing the NOE group to young controls (*P*>0.05) for all parameters tested [total dendritic field area, total dendritic length, Sholl analysis area under the curve]). There was also no significant difference in retinal ganglion cell dendritic morphology between retinal ganglion cells in the NOE group verses MOD or SEV, or between MOD and SEV groups (*P*>0.05 in all parameters tested) (Figures 3, 4).

### Retinal ganglion cell dendritic atrophy occurs in DBA/2J mice prior to significant optic nerve damage

**Figure 5:**
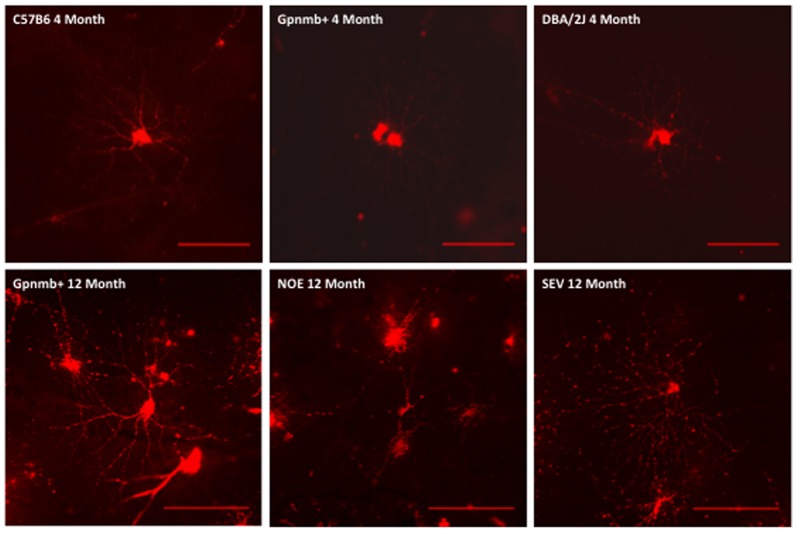
Representative panel of mouse retinal ganglion cells. Representative panel of mouse retinal ganglion cells from 4 month B6, D2-*Gpnmb^+^* and DBA/2J mice (*top row*) and 12 month D2-*Gpnmb^+^*, NOE and SEV DBA/2J mice (*bottom row*). Changes can be seen in the in dendritic morphology of NOE DBA/2J mice compared to age and sex matched D2-*Gpnmb^+^* mice. Scale bars  = 100 µm.

YFP is driven by the *Thy1* promoter in the transgenic mice and endogenous *Thy1* has been shown to be down-regulated in animal models of RGC damage or glaucoma [18], [27]. Therefore, it is possible that a down-regulation of *Thy1*-driven YFP expression would mask early dendrite changes in DBA/2J glaucoma. Therefore, in a second cohort of mice, we assessed dendrite morphology in RGCs that were labelled DiOlistically with the carbocyanine dyes DiI and DiO. These dyes distribute in the plasma membrane and can be used to label cells regardless of their physiological status [28]. Three strains of mice were used: 11–12 month DBA/2J mice (and their 4 month old, pre-disease counterparts), 11–12 month DBA/2J-*Gpnmb+* (D2-*Gpnmb^+^*) mice (and their 4 month old counterparts to serve as a control) and 4 month C57BL/6J (as an additional control). A representative panel of labelled cells is shown in Figure 5. RGC sub-classification is possible in the D2-*Gpnmb^+^* strains and we used this as a check against the possible of labelling bias. Using the Sun classification [39] we recorded the distribution of cell types as (%expected,% recorded) as A1 4.5(3.5), A2 5.5(7.1), B1 4.5(7.0), B2 9.0(5.9), B3 10.6(11.8), B4 5.7(8.2), C1 3.1(9.5), C2 10.2(3.5), C3 2.4(1.2), C4 5.9 (11.8), C5 11.2(16.5).

**Figure 6:**
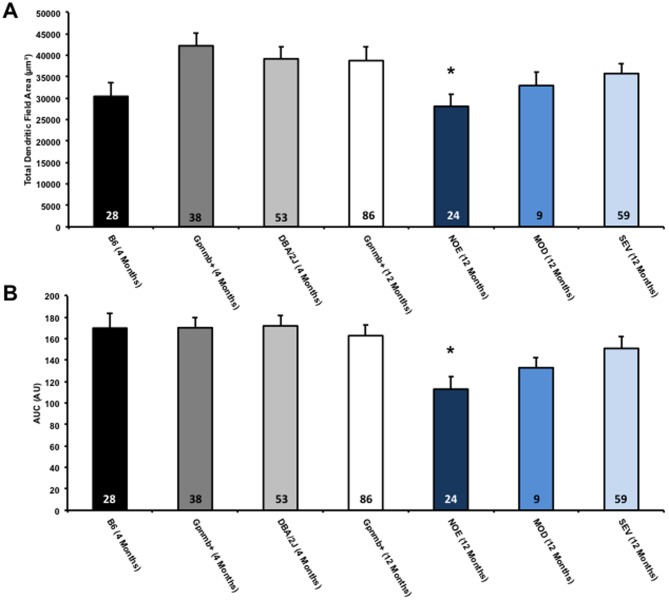
DiOlistically labelled no or early damage retinal ganglion cells show significantly decreased dendritic morphologies. Mouse retinas were DiOlistically labelled using carbocyanine dyes to observe the true changes to retinal ganglion cells in the DBA/2J mouse. There are significant reductions in total dendritic length, total dendritic field area (A) and Sholl analysis AUC (B) in NOE damage DBA/2J mice compared to age and sex matched Gpnmb+ mice. There were no changes to the dendritic morphologies of MOD and SEV damage retinal ganglion cells. Values for 4 month D2-*Gpnmb^+^* and NOE DBA/2J mice are shown. Additional C57BL/6J (B6) mice were used as a control for the D2-*Gpnmb^+^* mice (no significant differences in all parameters analysed). Error bars represent SEM. Numbers in bars represent the *n* of cells. *  = *P*<0.05.

**Figure 7:**
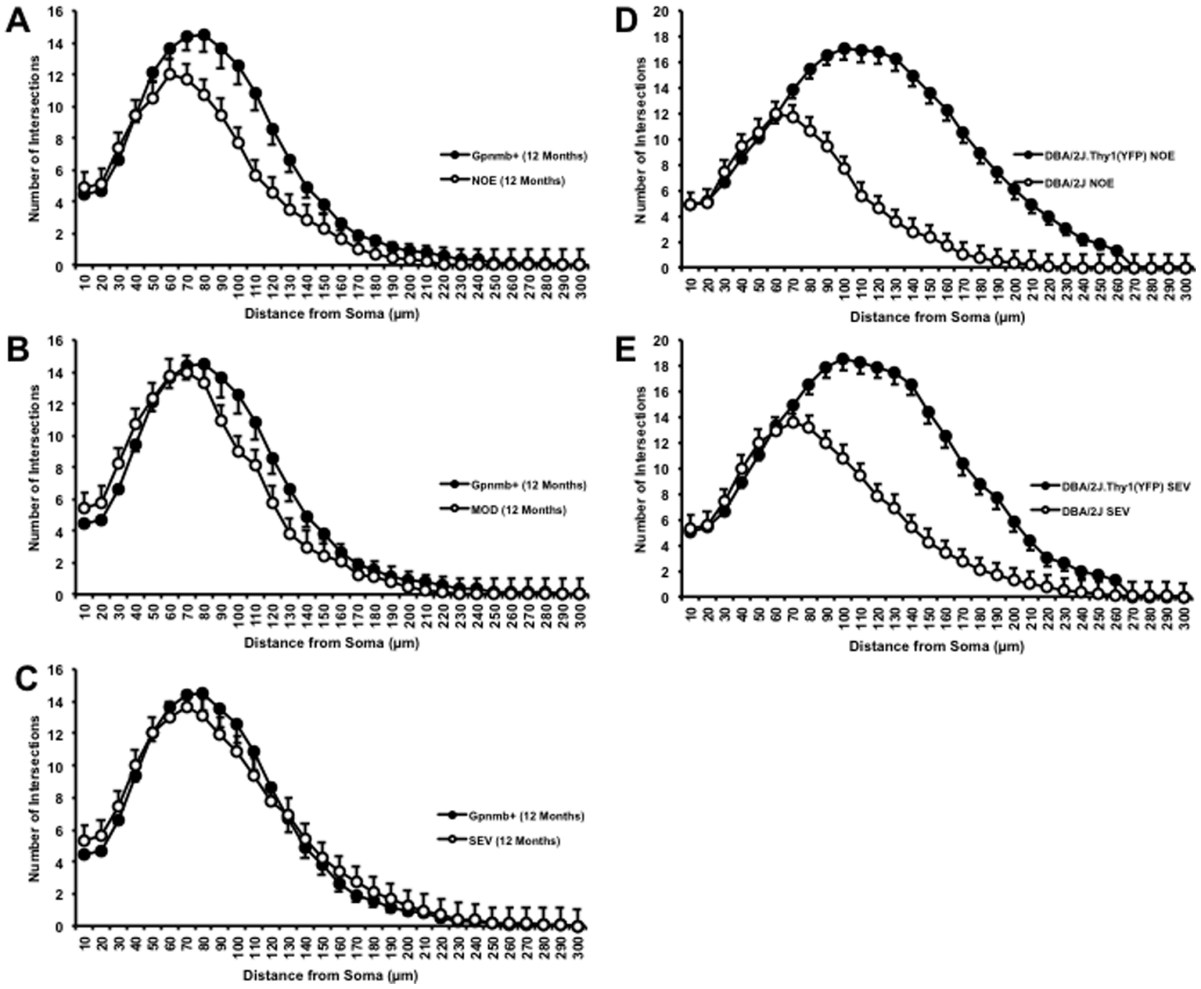
There is a significant shift in the Sholl analysis of DiOlistically labelled NOE DBA/2J mice but not MOD or SEV. There is a significant down and left-wards shift in the Sholl analysis of NOE compared to age and sex matched control D2-*Gpnmb^+^* mouse retinal ganglion cells representing a decrease in dendritic length as well as the number of branching points. (A) NOE v D2-*Gpnmb^+^*, (B) MOD v D2-*Gpnmb^+^*, (C) SEV v D2-*Gpnmb^+^*. Comparisons in Sholl analysis comparing the differences observed between strains (DiOlistically labelled DBA/2J and DBA/2J.Thy1(YFP)) for both mice with NOE (D) or SEV (E) optic nerve damage are shown. Error bars represent SEM.

**Figure 8:**
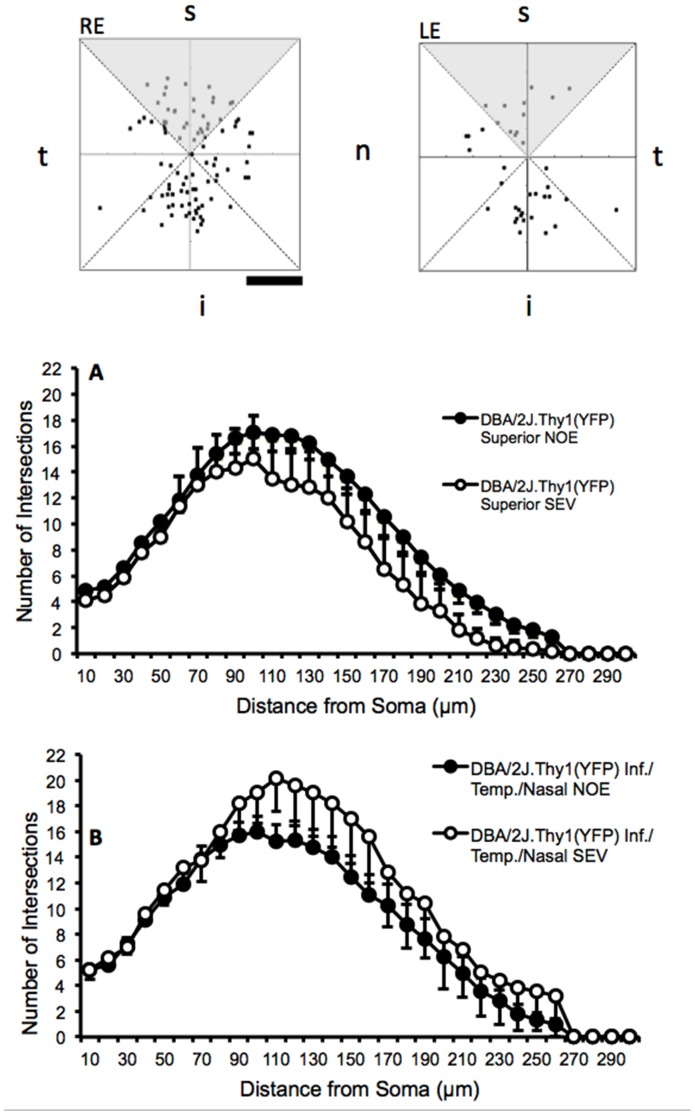
Retinal ganglion cell dendritic architecture is unchanged in DBA/2J.*Thy1*(YFP) mice independent of retinal eccentricity. Sectorial damage in the retina during glaucoma has been previously reported [8], [29], [30]. RGCs from superior, inferior, nasal and temporal quadrants were analysed separately. There was no significant difference in any of the parameters measured. *Top*; locations of labeled cells in DBA2J.*Thy1*(YFP) mice. RE: Right eye. LE: left eye. Scale bar 2 mm. (s,i,n,t: superior, inferior, nasal, temporal retinal segments centered on the optic nerve). Sholl plot (A) for RGC from superior retinas (shaded in panel) from eyes in which the optic nerve showed no apparent damage (NOE) filled circles (*n* = 27) or severe damage (SEV) open circles (*n* = 7). (B) For RGCs from inferior, nasal and temporal retinal segments in eyes in which the optic nerve showed no apparent damage (NOE) filled circles (n = 19), and severe damage (SEV) open circles (*n* = 6). Samples pooled from right and left eyes.

There was a significant decrease in dendritic architecture between DBA/2J eyes with no significant optic damage (NOE) and the D2-*Gpnmb^+^* age and sex match controls as assessed by total dendritic field area (D2-*Gpnmb^+^* [mean ±SEM/µm^2^], 36734±2238; NOE, 28097±2904, *P*<0.05), total dendritic length (D2-*Gpnmb^+^* [µm], 2173±122; NOE, 1462±136, *P*<0.0001) and Sholl analysis area under the curve (AUC) (D2-*Gpnmb^+^* [AU], 152±9; NOE, 113±12, *P*<0.05) (Figure 6, 7). There was no significant difference in dendritic morphologies between the MOD and SEV groups and the D2-*Gpnmb^+^* age and sex matched controls (*P*>0.05 in all parameters tested). No significant difference (*P*>0.05 in all parameters tested) was between any of the control groups; 4 month C57BL/6J vs 4 and 11–12 month D2-*Gpnmb^+^*. As sectorial damage in the retina during glaucoma has been shown independently by different groups [8], [29], [30], we addressed any bias in our analysis by analysing RGCs from superior, inferior, nasal and temporal quadrants separately. There was no significant difference in any of the parameters measured (Figure 8).

## Discussion

Dendritic pruning has been reported as a consistent feature of retinal ganglion cell damage in experimental glaucoma [10], [31]. However, previous studies were not able to determine the timing of dendritic changes relative to changes to other RGC compartments (such as axons). Here we report that RGC dendrite atrophy occurs at an early stage of glaucoma in DBA/2J mice. To our knowledge, this is the first study to show that RGC dendrite atrophy occurs prior to significant axon degeneration. Our data are consistent with previous reports of dendritic atrophy in this model [32]. The greatest reduction in area under the Sholl curves (compared to controls) was seen in RGCs from eyes with no or early (NOE) glaucoma. The Sholl curves showed less, but still significant change in moderate and severe glaucoma. Early dendritic atrophy was observed using DiOlistic labelling of RGCs but not in the DBA/2J.*Thy1*(YFP) mice. The differences in dendrite mapping are highlighted by comparison of the Sholl plots for YFP and Diolistics (Figure 6D,E). One explanation for this is that the YFP expression was reduced below detectable levels in RGCs prior to dendritic atrophy and that RGCs needed a degree of physiological health to express detectable levels of YFP. In support of this, endogenous *Thy1* has been shown to be down regulated in a number of studies involving mouse and rat glaucoma [18], [33] and has been used in other studies as a sensitive marker of RGC damage [13], [34], [35].

Our data are consistent with a pathophysiological model in which glaucoma induced dendritic atrophy occurs in RGCs with intact axons. In all labelled cells, axons could be traced to the optic nerve head. Also, these changes occurred in eyes with no significant optic nerve damage (no or early glaucoma based on PPD staining, assessed 1–2 mm behind the eye, see ***Methods***). Although axon damage was not assessed in all regions of the optic nerve, we have shown previously that earliest axonal changes occur near or close to the optic nerve head [8]. These observations are consistent with data in the primate glaucoma model in that dendritic atrophy occurs in moderate disease at stages when animals maintain visual field sensitivity.

The lack of significant dendritic atrophy in the eyes with more severe damage is of particular interest. Given the relatively small sample sizes for the Sholl analyses, we cannot exclude sample bias. However, a similar picture can be discerned with the YFP labelled RGCS though this did not reach significance. We cannot rule out the possibility that regional bias in Diolistic labelling could have targeted areas of the retina that were resistant to damage. However, this seems unlikely since retinal Diolistic labelling is generally diffuse [36] and at this stage of damage, most of the retina is affected [8]. It is also possible that differential RGC damage (i.e. class selective damage) has occurred. Jakobs *et al* investigated this possibility in the DBA2J model but could not find consistent differential sensitivity to damage in this model on the basis of RGC morphology [32]. The simplest explanation is that in the animals with severe damage surviving cells have relatively intact dendritic trees. Whether these cells have recovered from prior damage or were not damaged at any stage of the disease would require longitudinal analysis of RGC dendritic structure. Since intraocular pressure elevation in DBA/2J mice is often not maintained past 12 months of age it is possible that apparently intact RGC dendritic tree reflect a degree of recovery. Functional studies in rats, analysing changes in receptive field structure have suggest that this is possible [37]. Our results apply only to the analysis of dendritic trees since cell shrinkage has been reported previously in the DBA/2J mouse [32], [38] that precedes axon loss.

The early onset of dendritic damage in mice with no detectable morphological changes to axons in the optic nerve indicates that while changes may be initiated early at the level of the optic nerve head they can have significant effects throughout the cell. However, due to the resolution of the techniques used in this study, we cannot rule out that a very small number of axons in NOE optic nerves may be dying back. Our previous study assessed axon damage along the entire RGC axon extending from the cell body in the retina to the superior colliculus (SC) using a CFP fluorescent protein transgene that expresses in RGCs[8] (and discussed extensively in [39]). This study showed that during the NOE stage of glaucoma there is no evidence of dying back or disconnection in the brain, which occurs later [8], [39]. Agreeing with this, anterograde axon transport and connections in the brain are intact in all assessed NOE eyes ([25] and Figure 1). Given the random labelling of RGCs using DiOlistics it is extremely unlikely that there is a biased labelling for any rare cells with dying back axons. However, our data do not exclude the possibility that functional deficits in axon transport can occur at this early stage.

The maintenance and degeneration of dendritic arbours is a complex process that shows considerable variation throughout the CNS. Current evidence suggests limited scope for remodelling of dendrites and that dendritic pruning is complex. The underlying mechanism appears to depend on whether this is driven by injury of as part of development. The mechanisms underling dendritic atrophy remain unclear in glaucoma. Studies in experimental glaucoma have indicated that the retrograde transport of BDNF [40] may be compromised following the experimental elevation of intraocular pressure but this does not necessarily imply a direct link between a reduction in BDNF levels and the reduction in dendritic integrity. The exogenous administration of BNDF by intravitreal injection can result in the partial restoration of dendritic structure [41].

Atrophic changes in RGCs are likely to influence the response characteristics of RGCs. This has been explored in the primate glaucoma model using explant preparations which showed reduced contrast sensitivity [42]. More recently multi-electrode arrays have been used to explore the dimensions of the receptive field in an inducible mouse model of glaucoma which report an overall reduction in the size of the dendritic tree [30]. Changes in neuronal activity may influence the viability of RGCs and contribute to cell death. Both in adult and development the maintenance of dendritic integrity is dependent on the reservation of neuronal activity [43]. Electrophysiological recordings in experimental glaucoma have shown an overall reduction in neuronal activity. While a driver for this could be impaired summation within the RGC dendritic tree it does not rule out the possibility that a reduced rate of action potential could result in impaired endogenous production of neurotrophic agents (for a review see [44]).

Several factors are likely to conspire in dendritic degeneration. Histological studies in human glaucoma have confirmed the presence of varicosities within the axons of retinal ganglion cells [45] which are most likely a result of the change in the mitochondrial network in which mitochondria assume a more discrete configuration. Mitochondria are known to be critical to synaptic maintenance and a change in distribution or a reduction in number is likely to have widespread effects on neuronal function. Fragmentation of the mitochondrial network is a common feature of neuronal distress and has been observed in conditions in which mitochondrial dysfunction (*e.g.* in *Opa1* mutants) in which dendritic atrophy and mitochondrial beading is a hallmark of early disease [36], [46]. The convergence of multiple pathological drivers may be significant in explaining the role of some of the risk factors in glaucoma, in particular ageing which renders the retina more susceptible to damage; possibly as a result of reduced mitochondrial efficiency and lowered resistance to external stressors [47]. Defective mitochondrial transport has been detected in experimental rat glaucoma [48] possibly as a result of defective intracellular motility [49] and this could underpin some of the degenerative changes since mitochondria are important for the maintenance of synaptic contacts and dendritic trees [46], [50], [51].

These considerations do not rule out the role of exogenous (non-RGC related factors). Macrophage activity, in the form of microglial activation and migration, is an early feature of both experimental and human glaucoma [52]. While there is little doubt that this is a driver of optic nerve and axonal damage, microglia are also activated within the retina and inner plexiform layer where they can play a direct role in the elimination of synapses and changes to dendritic structure [53]. RGC synaptic pruning, possibly mediated by the complement cascade, has also been suggested to occur early in glaucoma and may precede dendritic atrophy [18].

## Materials and Methods

### Mouse strain, breeding and husbandry

Mice were housed in a 14 h light/10 h dark cycle as previously described with food and water available *ad libitum*. All breeding and experimental procedures were undertaken in accordance with the Association for Research for Vision and Ophthalmology Statement for the Use of Animals in Ophthalmic and Research. The Institutional Biosafety Committee (IBC) and the Animal Care and Use Committee (ACUC) at the Jackson Laboratory approved this study. The DBA/2J, DBA/2J-*Gpnmb+*, and DBA/2J.*Thy1*(YFP) strains have been described in detail elsewhere. C57BL/6J mice from The Jackson Lab facility (Bar Harbor, ME) were used as an alternative age matched control. All mice were female. We used D2-*Gpnmb^+^* mice, a non glaucomatous substrain of DBA/2J that carry a wild type version of the *Gpnmb* gene responsible for the development of the glaucomatous phenotype. Although these animals develop mild pigment dispersion from the iris as a result of a mutation in the Tyrp1b gene this is not associated with elevated IOP [54].

### Anterograde axon transport

Thirty 10–12 months old DBA/2J-*Gpnmb+*, and DBA/2J were injected with 1–2 µl (1 mg/ml) Alexa Fluor 594-Cholera Toxin subunit B conjugate (Invitrogen) into the vitreous using a Hamilton syringe (35-gauge needle). After 48 to 72 hours they were anesthetized and euthanized via 4% PFA cardiac perfusion. Brains were submersion fixed for 24 hours after perfusion, cryoprotected in 30% sucrose overnight, OCT cryoembedded, and sectioned at 50 µm. Alexa Fluor 594 was visualized using an SP5 confocal microscope (Leica). The entire SC was assessed.

### Retinal preparation

4 and 12 month female C57BL/6J, DBA/2J-*Gpnmb+*, and DBA/2J mice (C57BL/6J, 4 months *n* = 3; DBA/2J-*Gpnmb+*, 4 months *n* = 3, 12 months *n* = 7; DBA/2J, 4 months *n* = 3, 12 months *n* = 13) were killed by cervical dislocation and the eyes quickly enucleated and placed in Neurobasal -A media (Invitrogen) at RT. The eyes were punctured at the limbus and a slit cut in the sclera to remove the cornea and sclera anterior to the ora serrata, along with the lens and vitreous. Three cuts were made in the retina before it was flat-mounted ganglion cell layer up on a cell culture insert (Millipore) and submerged Neurobasal –A media. Retinas were incubated at 37°C and 4% CO_2_ ready for DiOlistic labelling using a gene gun. The total time between death and DiOlistic labelling was less than 10 minutes.

1–3 month (*n* = 7) and 9.5–11 month female DBA/2J.*Thy1*(YFP) mice (*n* = 16) were killed by cervical dislocation, the eyes quickly enucleated and placed into 4% PFA at RT for 1 h. Retina were removed and dissected as above, mounted onto slides, coverslipped and sealed under Floromount (Sigma Aldrich) ready for imaging.

### DiOlistic labelling using the gene gun

Bead delivery and preparation of DiOlistic labelling of retinal ganglion cells has been described in detail elsewhere [24], [36], [55]. Briefly, 100 mg of tungsten particles (1.7 µm; Bio-Rad) was placed in a thin even layer on a clean, glass slide. DiI or DiO (80 mg, Invitrogen), was then mixed in 800 µl of methylene chloride, and poured over the tungsten particles. The methylene chloride evaporated quickly to leave DiI or DiO coated tungsten particles, which were then transferred onto clean wax-paper or tinfoil. This powder was then funnelled into a length of 1.4 mm ‘Gold-Coat’ tubing (Bio-Rad) and allowed to settle, resulting in a light application of the powder upon the inside of the tubing. Excess powder was funnelled off and the tubing was cut into 1.2 cm lengths for storage in the dark at room temperature ready for use.

Retinas were shot once at 120 psi using a Helios gene gun (Bio-Rad) from 5 cm with a 3.0 µm pore size, high pore density, cell culture insert (Becton Dickinson) to block the passage of aggregated tungsten particles. Retinas were then incubated for 30 min to facilitate dye diffusion before being placed in 4% PFA at RT for a further 30 min. Retinal preparations were then mounted retinal ganglion cell side up and coverslipped under Floromount and sealed with nail polish. Images were taken within 48 hours.

### Retinal ganglion cell dendritic morphological analysis

Image stacks (0.1 µm slice width) of 400 RGCs (C57BL/6J, 4 months *n* = 28; DBA/2J-*Gpnmb+*, 4 months *n* = 38, 12 months *n* = 86; DBA/2J, 4 months *n* = 53, 12 months *n* = 92; DBA/2J.*Thy1*(YFP), 1–3 month *n* = 35, 9.5–11 month *n* = 68) were obtained with a Zeiss LSM 510 confocal microscope (Carl Zeiss) or Leica TCS SP5 confocal microscope (Lieca) using a 20× objective to allow the capture of the entire dendritic tree within a single image. Dendritic morphologies were analysed using ImageJ to measure dendritic field area (measured using the convex polygon tool to join the outer most points of the dendritic tree), an ImageJ plugin, NeuronJ to measure total dendritic length, and a custom Matlab macro to run a Sholl analysis [56], [57].

### Axon labelling with PPD and grading of glaucomatous damage

The processing of optic nerves and staining with paraphenylenediamine (PPD) which darkly stains the axoplasm and myelin sheath of dying axons has been reported previously [8], [15], [58]. *In brief*, intracranial portions of optic nerves were fixed in 4% PFA at RT for 48 h, processed and embedded in plastic. A segment of optic nerve from within a region up to 1 mm from the posterior surface of the sclera was sectioned (1 µm thick sections) and stained with PPD. Typically 30–50 sections are taken from each nerve. Homology between sections is considered during grading. Optic nerves were analysed and given 1 of 3 gradings [59]:

1. No or early damage (NOE) – less than 5% axons damaged. This level of damage is seen in age and sex matched non-glaucomatous mice,
2. Moderate damage (MOD) – average of 30% axon loss,
3. Severe (SEV) – >50% axonal loss and damage.
